# A Case of Marburg Variant Multiple Sclerosis Treated With Rituximab

**DOI:** 10.7759/cureus.90698

**Published:** 2025-08-21

**Authors:** Melanie Khamlong, Aishwarya Saripalli, Noah Yan, Anthony Bettencourt, Katayoun Sabetian

**Affiliations:** 1 Internal Medicine, University of California, Los Angeles (UCLA) - Kern Medical, Bakersfield, USA; 2 Neurology, University of California, Los Angeles (UCLA) - Kern Medical, Bakersfield, USA

**Keywords:** fulminant multiple sclerosis, immunosuppressant therapy, marburg variant, multiple sclerosis, rituximab

## Abstract

Marburg variant is a rare, monophasic form of multiple sclerosis (MS) characterized by an aggressive clinical course. Diagnosis is confirmed with brain MRI demonstrating multiple focal T2-hyperintense lesions and with histopathology. While there is no established standard of care, high-dose corticosteroids followed by immunosuppressive therapies have commonly been used. Rituximab has been reported in the treatment of two previously described cases. We report a case of a 59-year-old woman diagnosed with Marburg variant MS who was successfully treated with rituximab.

## Introduction

Marburg variant is a rare, monophasic form of multiple sclerosis (MS) characterized by an aggressive clinical course [1]. Patients may present with headache, confusion, gait instability, visual disturbances, and hemiparesis. The disease often progresses rapidly, leading to severe disability or death within weeks to months. Brain MRI typically demonstrates multiple focal T2-hyperintense lesions of varying sizes [2]. Histopathological examination reveals extensive macrophage infiltration, widespread demyelination, hypertrophic astrocytes, and significant axonal damage [2].

While there is no established standard of care, high-dose corticosteroids followed by immunosuppressive therapies have commonly been used. Rituximab has been reported in the treatment of two previously described cases [3,4]. We report a case of a 59-year-old woman diagnosed with Marburg variant MS who was successfully treated with rituximab.

## Case presentation

A 59-year-old Spanish-speaking lady hairdresser with diabetes mellitus initially presented to the emergency department with six weeks of progressive forgetfulness and a 20-pound weight loss over six months. Her family reported two near-miss driving incidents, one involving veering off the road and another nearly colliding with an adjacent car. They also noted increased forgetfulness, including missing appointments to pick up her grandchildren and neglecting meal preparation.

**Figure 1:**
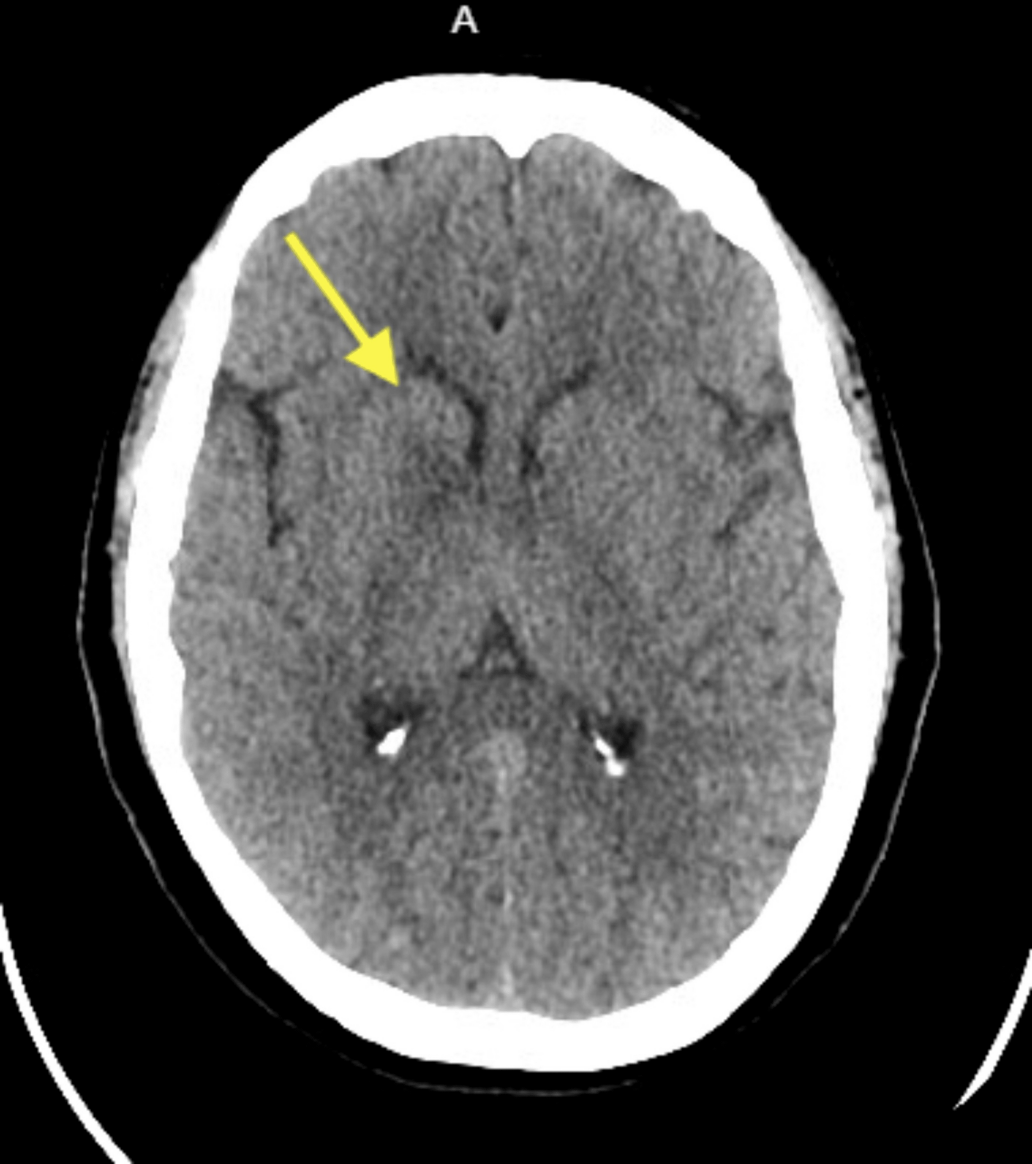
Non-contrast head CT revealing a right basal ganglia lacunar hypodensity suspicious for an acute infarct (yellow arrow).

**Figure 2:**
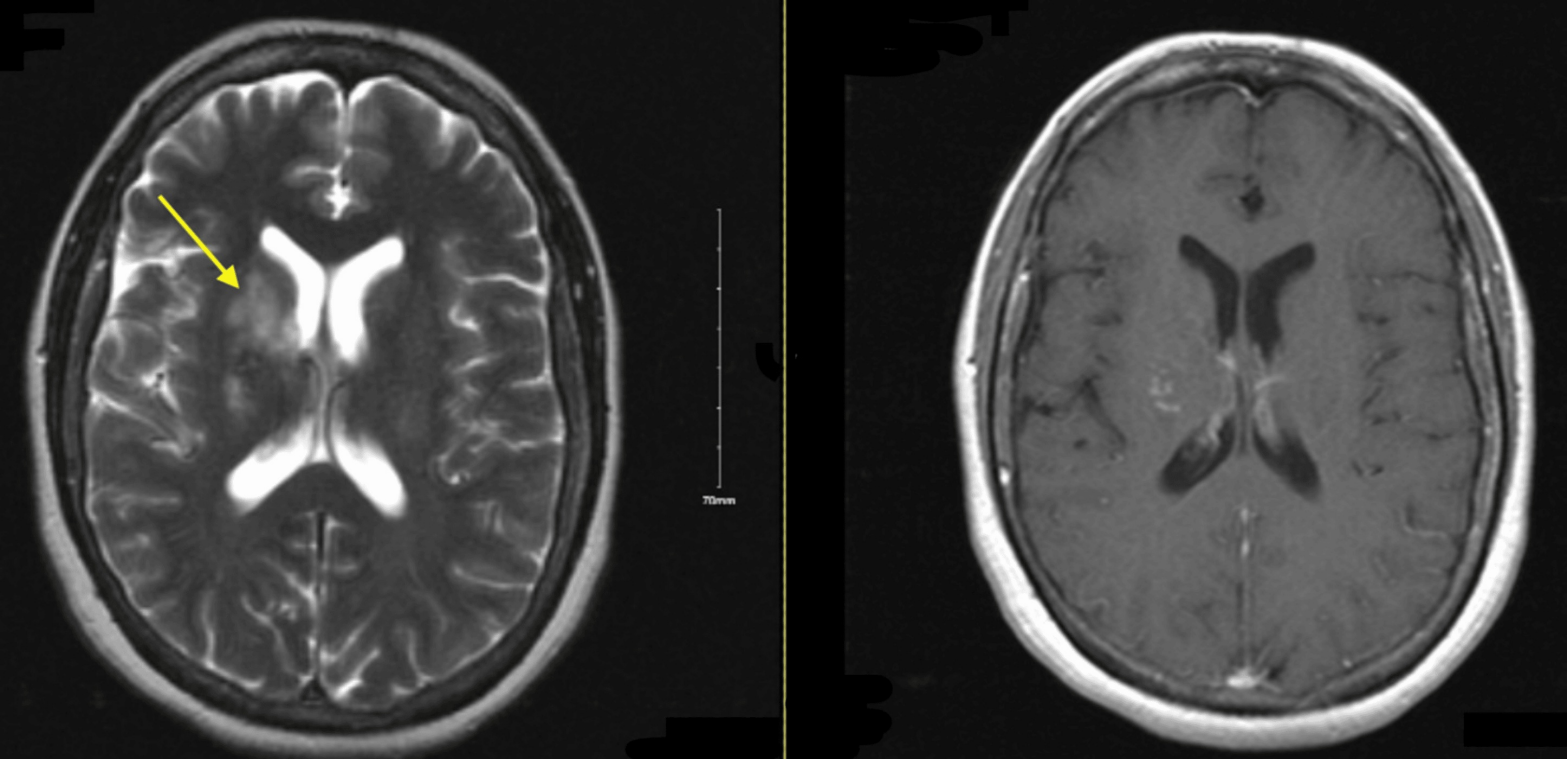
Initial brain MRI and T1 with gadolinium. Left image shows initial brain MRI with and without contrast demonstrating right basal ganglia T2/FLAIR hyperintensity (yellow arrow) extending across the midline to the left, with slight enhancement in the right thalamus and periventricular areas. Right image shows T1 with gadolinium. FLAIR: fluid-attenuated inversion recovery.

**Figure 3:**
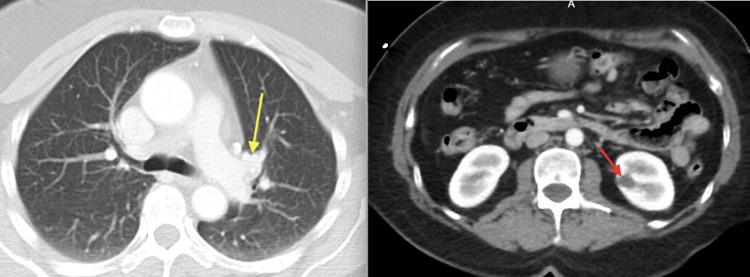
CT of the chest and abdomen. CT of the chest (left) demonstrates old left hilar calcifications (yellow arrow), and CT of the abdomen (right) demonstrates a benign renal cyst (red arrow), with no evidence of malignancy.

**Figure 4:**
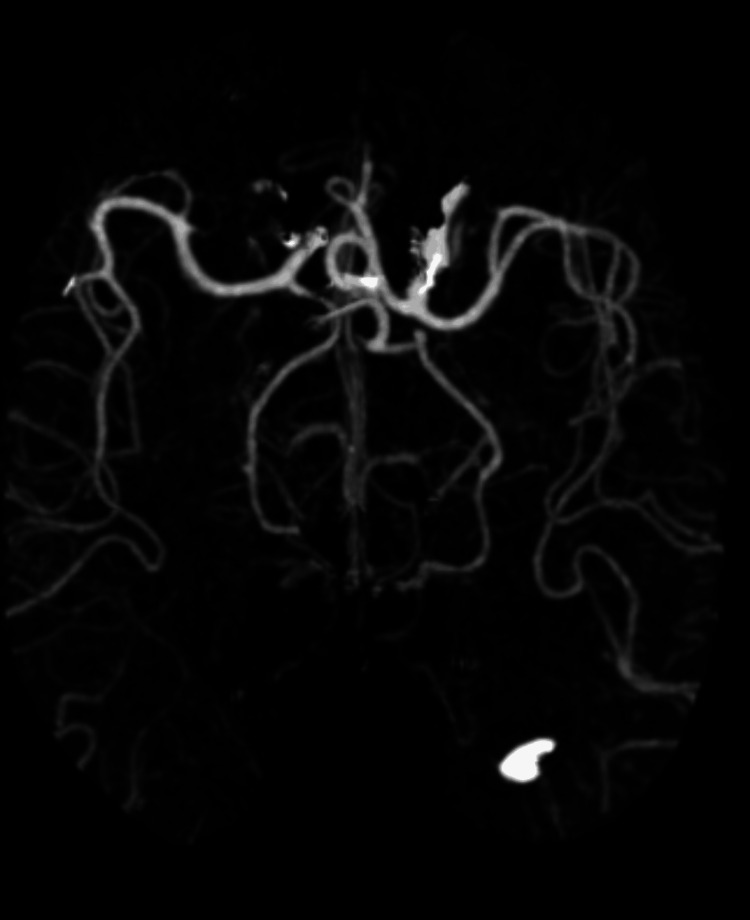
CT angiography of head and neck which demonstrated no significant stenosis or occlusion of major arteries.

Neurologic examination showed she was alert and oriented to name, place, and time, with muscle strength 5/5 in all extremities, intact sensation throughout, absent reflexes, and a steady gait with mild difficulty on tandem walking. Her Mini-Mental Status Exam (MMSE) score was 29/30, with recall of one out of three words [5]. A non-contrast head CT revealed a right basal ganglia lacunar hypodensity suspicious for an acute infarct (Figure 1), prompting admission for stroke evaluation, and she was started on aspirin and clopidogrel. Brain MRI with and without contrast demonstrated right basal ganglia T2/fluid-attenuated inversion recovery (FLAIR) hyperintensity extending across the midline to the left, with slight enhancement in the right thalamus and periventricular areas (Figure 2). Additional workup, including CT angiography of the head and neck and CT of the chest, abdomen, and pelvis, was unremarkable apart from old left hilar calcifications and a benign renal cyst, with no evidence of malignancy (Figures 3, 4). Infectious evaluation (HIV, syphilis, ESR, CRP, coccidioidomycosis serology, QuantiFERON-TB) was negative (Tables 1-3). Aspirin and clopidogrel were discontinued. Given recent anti-platelet use, she was scheduled for outpatient lumbar puncture (LP) and neurosurgery consultation. She was discharged home.

**Table 1:** Initial lumbar puncture with results of CSF studies, including both infectious serology and autoimmune encephalitis panel. CSF: cerebral spinal fluid, NMDAR: N-methyl-D-aspartate receptor, LGI1: leucine-rich glioma-inactivated 1, GAD65: glutamic acid decarboxylase 65, GABA-B-R: gamma-aminobutyric acid-B receptor, CASPR2: contactin-associated protein-like 2, AMPAR: alpha-amino-3-hydroxy-5-methyl-4-isoxazolepropionic acid receptor, GluR2: glutamate receptor 2.

CSF Workup	Value	Reference
Opening pressure	10 cm H_2_O	Normal: 6-25 cm H_2_O
WBC count	4	Normal high: >5/µL
CSF neutrophil %	8	
CSF lymphocyte %	85	
CSF monocyte %	6	
CSF basophil %	1	
Glucose	68	Normal range: 40-75 mg/dL
Protein	34	Normal range: 15-45 mg/dL
CSF cocci serology	Non-reactive	Non-reactive
NMDAR CSF	Negative	Negative
LGI1	Negative	Negative
GAD65 CSF	Negative	Negative
GABA-B-R	Negative	Negative
CASPR2	Negative	Negative
AMPAR (GluR2)	Negative	Negative

**Table 2:** Serum infectious disease diagnostic workup.

Infectious Workup	Result	Reference
HIV 1, 2 antigen/antibody	Non-reactive	Non-reactive
Syphilis Ab qualitative	Non-reactive	Non-reactive
Hepatitis C antibody	Non-reactive	Non-reactive
QuantiFERON Gold	Negative	Negative
Cocci serology	Non-reactive	Non-reactive

**Table 3:** Serum autoimmune diagnostic workup. ANA: antinuclear antibody, ANCA: antineutrophil cytoplasmic antibody.

Autoimmune Workup	Value	Reference
ANA screen	Positive	Negative
ANA titer	1:80	Normal: <1:40
ANA pattern	Nuclear, homogenous	
ANCA Screen	Negative	Negative

**Figure 5:**
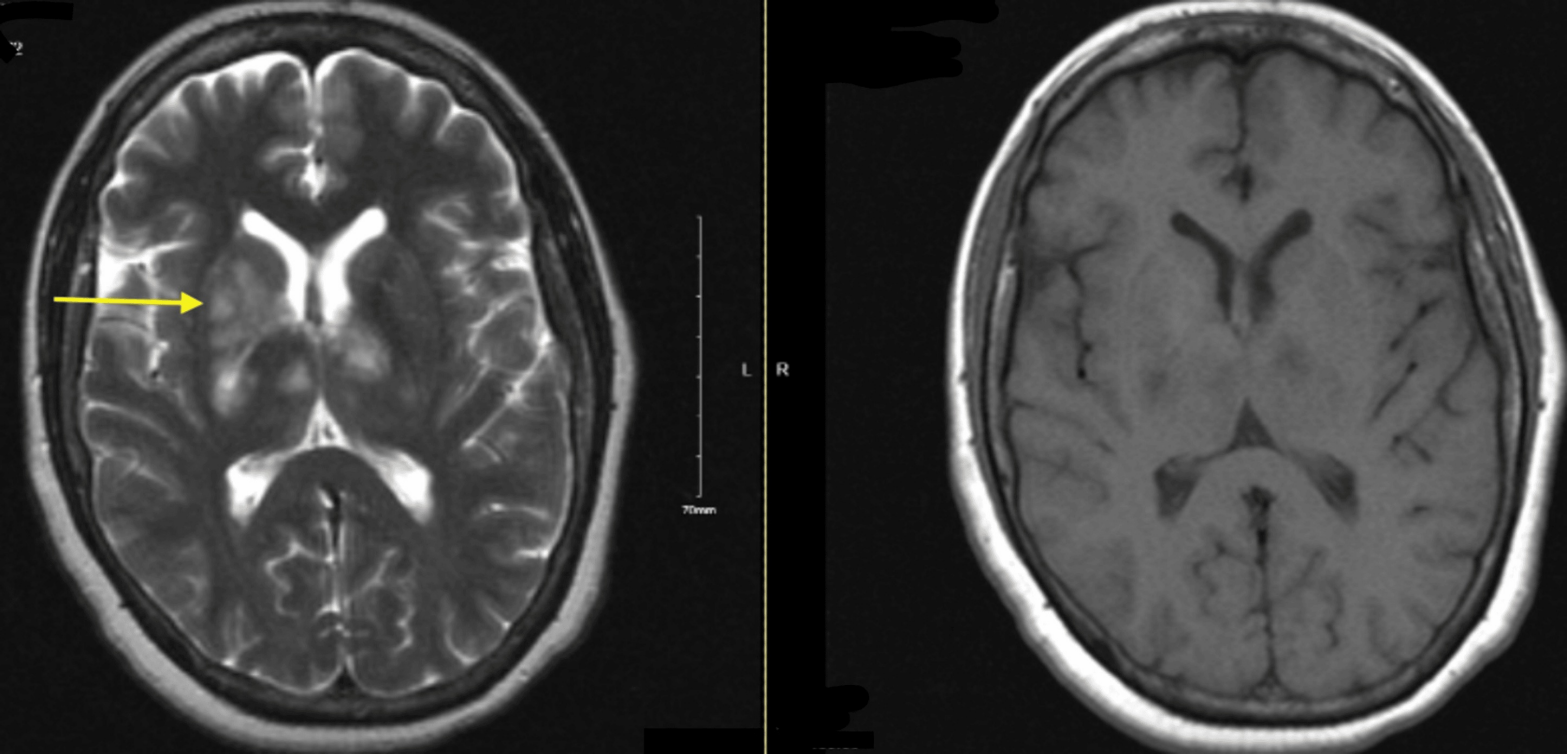
Repeat brain MRI and T1 with gadolinium. Repeat brain MRI showed mildly increased bilateral thalamic FLAIR/T2 signal and an enlarged right basal ganglia lesion (4.3 x 1.7 cm) (left image, yellow arrow). Right image shows T1 with gadolinium. FLAIR: fluid-attenuated inversion recovery.

Outpatient LP showed opening pressure of 10 cm H₂O, WBC 4 (85% lymphocytes), glucose 68, and protein 34; autoimmune encephalitis panel was negative. One week later, she was readmitted with worsening memory, impaired left-hand coordination, gait disturbance, and new urinary incontinence. The family reported a fall in the bathroom and bedwetting. On exam, she was drowsy but arousable, oriented to the date and hospital, with intact strength (5/5) and sensation in all extremities. Repeat brain MRI showed mildly increased bilateral thalamic FLAIR/T2 signal and an enlarged right basal ganglia lesion (4.3 x 1.7 cm) (Figure 5). Concern for malignancy prompted transfer to higher level of care for brain biopsy.

At the outside facility, repeat LP revealed WBC 4, RBC 2, protein 36, negative flow cytometry and cytology, and three oligoclonal bands, normal IgG synthesis and index. EEG demonstrated left hemispheric slowing without epileptic discharge. She received five days of intravenous methylprednisolone (1 g/day), after which motor strength declined to 4/5 in the left arm and leg. Follow-up MRI showed lesion expansion, change in pattern of enhancement, and a new left subcortical white matter T2 lesion. She underwent stereotactic brain biopsy and was transferred back to our hospital.

On return, she was drowsy, oriented to hospital and year, but not month nor date and had paralysis of left arm and leg with 0/5 strength. Brain biopsy results few days later revealed demyelination with CD5+ lymphocytes, macrophages, and perivascular inflammation, findings suggestive of Marburg’s variant of multiple sclerosis. After multidisciplinary consultation with neuroimmunologist, she was started on rituximab 1 g intravenous. Five days later, left hand grip and foot dorsiflexion improved. A second rituximab dose was given two weeks later. She was eventually discharged home with family and able to ambulate with a walker.

**Figure 6:**
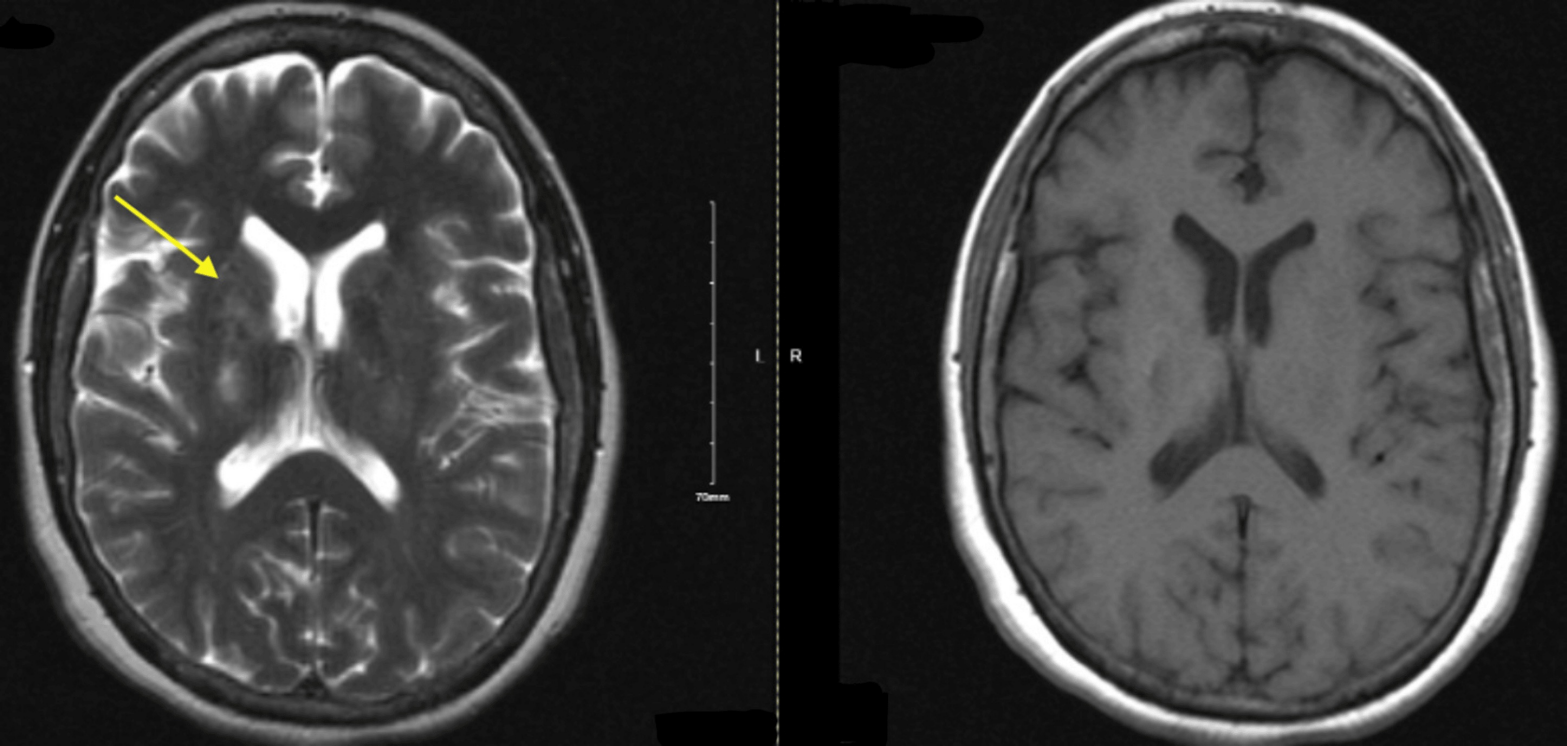
Repeat MRI and T1 with gadolinium. Repeat MRI, performed three months after the second Rituximab dose, showed improvement in lesions involving the right periventricular region, basal ganglia, bilateral thalami, and the posterior limb of the right internal capsule (left image, yellow arrow). Right image is T1 with gadolinium.

At neurology follow-up seven weeks later, she reported resolution of left-sided weakness and walked independently, although family noted persistent forgetfulness. Repeat MRI, performed three months after the second rituximab dose, showed improvement in lesions involving the right periventricular region, basal ganglia, bilateral thalami, and the posterior limb of the right internal capsule (Figure 6). A third rituximab infusion was given six months after her last dose. The patient returned to working as a hairdresser and was driving without difficulty, occasionally misplacing her key or purse.

## Discussion

Marburg variant is a rare and aggressive form of multiple sclerosis (MS), accounting for less than 4% of MS cases [6]. Patients typically present with rapidly progressive neurological symptoms such as weakness, hemiparesis, confusion, gait disturbance, and visual impairment. The disease often leads to significant disability or death within weeks to months. Diagnosis can be challenging and generally requires brain biopsy confirmation. Histopathological examination reveals extensive macrophage infiltration, widespread demyelination, hypertrophic astrocytes, and significant axonal damage [2]. Diagnostic workup often includes MRI brain, which typically shows multiple focal T2 lesions of varying sizes, which may coalesce to form large white-matter plaques disseminated throughout hemispheric white matter and brainstem [2]. In most reported cases, biopsies were performed after patients showed clinical deterioration despite initial treatment efforts.

Due to its fulminant course, prompt and aggressive therapy is essential. However, no standardized treatment guidelines exist for Marburg variant MS. Most cases have been initially managed with high-dose intravenous corticosteroids, with escalation to plasma exchange if clinical improvement was not observed [7]. Despite these treatment modalities, many patients continued to decline or experienced disease relapse.

In light of these outcomes, immunosuppressive therapies have been employed in most cases [8]. Cyclophosphamide and mitoxantrone are the most frequently used agents, likely due to their effects in suppressing T- and B-cell proliferation. Reports of cyclophosphamide use generally describe improvements in neurological disability [4,9-11]. Mitoxantrone has been associated with either clinical remission or significant improvement in clinical status [12-14]. However, the potential adverse effects of these agents, such as cardiotoxicity and myelosuppression, necessitate careful monitoring.

Rituximab, a humanized chimeric anti-CD20 monoclonal antibody widely used in B-cell malignancies and immune-mediated disorders, has only been described in two reported cases of Marburg variant of MS [15]. In one case, a patient with relapsing-remitting MS was initially treated with natalizumab for visual and cognitive symptoms but required escalation to fingolimod due to worsening cognitive function. Rituximab was subsequently added, resulting in no further relapses or new MRI lesions [3]. Another case described a patient initially treated with Cyclophosphamide who, after one year, developed new T2-hyperintense lesions and was transitioned to rituximab [4].

Our patient received induction therapy with two doses of rituximab administered two weeks apart. At her neurology follow-up approximately two months later, she demonstrated complete resolution of left-sided hemiplegia, and after a third infusion six months later, her memory had improved, allowing her to return to work and driving. Given this favorable response in our patient, rituximab may present an effective treatment option for Marburg variant MS. However, further research is necessary to directly compare the efficacy and safety of available immunosuppressive therapies for this rare and devastating disease.

## Conclusions

We report a case of Marburg variant multiple sclerosis in a patient who initially presented with confusion, followed by progressive gait disturbance and left hemiplegia. The diagnosis was confirmed through brain MRI and biopsy. After worsening of symptoms despite high-dose methylprednisolone, she was started on induction therapy with rituximab, receiving two doses two weeks apart. At her two-month follow-up after hospital discharge, she demonstrated complete resolution of left-sided hemiplegia, and six months later, her memory deficit almost completely resolved. Given this favorable outcome, rituximab, which depletes B cells, may be a promising therapeutic option for patients with Marburg variant MS, which is mainly a T-cell-mediated disease.
